# Lymphome de hodgkin primitif de la thyroïde: à propos d’un cas

**DOI:** 10.11604/pamj.2017.28.266.13736

**Published:** 2017-11-24

**Authors:** Halima Hadri, Salma Fares, Tarek Moutiqui, Ghizlane Lembarki, Nadia Moussali, Naima El Benna, Meriem Regragui, Nisrine Bennani, Sanaa Naoumi, Mehdi Karkouri, Asmaa Quessar

**Affiliations:** 1 Service d’Hématologie et d’Oncologie Pédiatrique, Hôpital 20 Août 1953, CHU Ibn Rochd, Casablanca, Maroc; 2 Service de Radiologie de l’Hôpital 20 Aout 1953, CHU Ibn Rochd, Casablanca, Maroc; 3 Service Central d’Anatomie Pathologique, CHU Ibn Rochd, Casablanca, Maroc

**Keywords:** Lymphome de hodgkin, extra-nodal, thyroïde, Hodgkin's lymphoma, extra-nodal, thyroid

## Abstract

Les lymphomes thyroïdiens primitifs sont une entité clinique rare, qui ne dépassent pas 5% des lymphomes diagnostiqués, se produisent plus fréquemment chez les femmes que chez les hommes, avec un pic d'incidence à la sixième décennie de la vie. La relation avec une thyroïdite chronique est bien connue. Le sous type hodgkinien, encore plus rare, peu décrit dans la littérature; posant un problème diagnostique. La confirmation diagnostique est portée le plus souvent sur la pièce opératoire. Pour mieux comprendre cette entité, nous rapportons le cas d'un patient de 64 ans, sans notion de thyroïdite chronique, admis pour un lymphome de Hodgkin de la thyroïde, diagnostiqué sur une masse cervicale antérieure. La thyroïdectomie avec études histopathologique et d'immunohistochimique avaient confirmé le diagnostic. Le patient avait reçu une chimiothérapie de type ABVD (Adriblastine-Bléomycine-Vinblastine-Dacarbazine) et programmé pour une radiothérapie.

## Abstract (en)

Primary thyroid lymphoma is a rare clinical entity, which does not exceed 5% of the diagnosed lymphomas, occur more frequently in women than in men, with a peak incidence in the sixth decade of life. The relationship with chronic thyroiditis is well known. The Hodgkin subtype even rarer; little described in the literature; Posing a diagnostic problem. Diagnostic confirmation is usually carried out on the surgical specimen. To better understand this entity, we report the case of a 64-year-old patient, with no notion of chronic thyroiditis, admitted for Hodgkin's lymphoma of the thyroid, diagnosed on an anterior cervical mass. Thyroidectomy with histopathological and immunohistochemical studies confirmed the diagnosis. The patient had received chemotherapy type ABVD (Adriblastin-Bleomycin-Vinblastine-Dacarbazine) and programmed for radiotherapy.

## Introduction

Le lymphome de Hodgkin (LH) est une maladie lymphoproliférative maligne, caractérisée par la présence de cellules de reed-sternberg (CRS) et leur infiltration inflammatoire. Il présente 8.2% de tous les lymphomes diagnostiqués [1] avec une incidence annuelle d'environ 3 nouveaux cas pour 100.000 habitants [2]. Le LH se présente habituellement par des adénopathies périphériques indolores (70%), le plus souvent de localisation cervicale (60-80%), la localisation extranodale des LH est moins fréquente que dans les lymphomes non hodgkiniens (LNH), représente environ 5% à 10% des cas [3]. La localisation thyroïdienne du LH est encore plus rare, occupant le quatrième rang des lymphomes thyroïdiens primitifs (LTP), qui ne présentent quant à eux que près de 5% de tous les lymphomes diagnostiqués [4], dans la majorité des cas; les LTP sont de type non hodgkinien avec une prédominance du lymphome diffus à grande cellules B jusqu'à 70% suivi du lymphome B de la zone marginale (MALT) dans environ 23% , tandis que le LH thyroïdien ne représente que 7% des LTP [5, 6]. Selon la littérature, il y a une plus grande incidence des lymphomes thyroïdiens dans les cas de thyroïdite auto-immune [7], notamment la thyroïdite Hashimoto (TH), connue comme un facteur de risque pour le développement de LTP [8], elle induit une prolifération lymphocytaire dans la glande thyroïde dépourvue de lymphocytes. La prise en charge des LTP ne diffère pas de celle des lymphomes nodulaires, ainsi pour le LH thyroïdien comme pour le LH nodulaire le traitement repose sur une polychimiothérapie systémique associée ou non à une radiothérapie, le recours à la chirurgie n'est pas indiqué. Nous rapportons le cas d´un patient diagnostiqué avec un lymphome de hodgkin thyroïdien, pour mieux comprendre cette entité.

## Patient et observation

**Figure 1:**
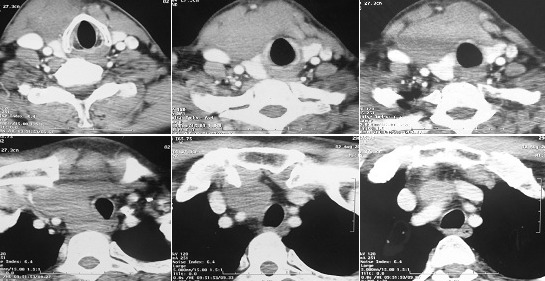
TDM initial

Un homme âgé de 64 ans, sans antécédents pathologiques notables, notamment pas de notion de dysfonction thyroïdienne, qui avait été admis au service de chirurgie de l'oto-rhino-laryngologie (ORL), pour prise en charge chirurgicale d'une masse cervicale antérieure, la symptomatologie semble remonter à 8 mois auparavant par l'installation d'une tuméfaction de la région cervicale antérieure augmentant progressivement de volume, occasionnant une dysphonie avec un prurit et des sueurs nocturnes, sans notion de fièvre ni d'amaigrissement chiffré. L'examen physique initial retrouvait un patient en assez bon état général; une masse cervicale antérieure ferme, indolore, de 7 cm de grand axe avec un empâtement du creux sus-sternal, sans adénopathies périphériques. Une TDM cervicale initiale avait montré une masse tissulaire de la loge thyroïdienne latéralisée vers la droite de 70x40 mm, se plongeant au niveau du médiastin moyen et antérieur, avec de petites adénopathies latéro-cervicale et sous-claviculaire gauches (Figure 1). Au décours de l'acte opératoire; il avait été mis en évidence une masse dure infiltrant les muscles sous-hyoïdiens, se plongeant en endothoracique, probablement développée au dépend de l'isthme thyroïdien, la chirurgie avait consisté sur une exérèse incomplète de la masse qui avait été coupé au niveau thoracique, suivie d'une lobectomie gauche puis droite. L'étude histologique de la pièce chirurgicale avait montré au niveau de l'isthme thyroïdien une prolifération tumorale dissociée par d'épaisses travées de fibrose collagéinisée et montre une prolifération tumorale lymphogranulomatose, faite de cellules d'allure hodgkinienne par place, de cellules de Reed-Sternberg typiques, mêlées à des lymphocytes, des plasmocytes, des granulocytes et des histiocytes avec présence également de foyers de nécrose fibrinoide par endroit. Le parenchyme thyroïdien au niveau des lobes droit et gauche était dystrophique, sans signe de malignité. L'étude immuno-histoichimique avait montré une intense et diffuse positivité des cellules tumorales aux anticorps anti-CD15, anti-CD30; la cytokératine, le CD3 et le CD20 étaient négatifs, concluant ainsi pour un lymphome hodgkinien de type scléro-nodulaire de l'isthme thyroïdien.

**Figure 2:**
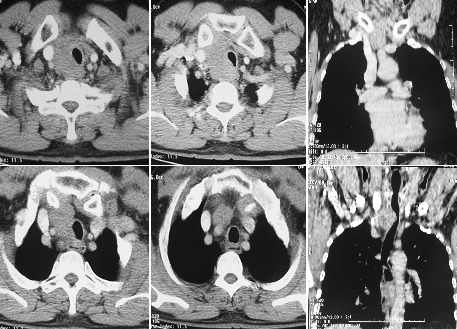
TDM de rechute après chirurgie

**Figure 3:**
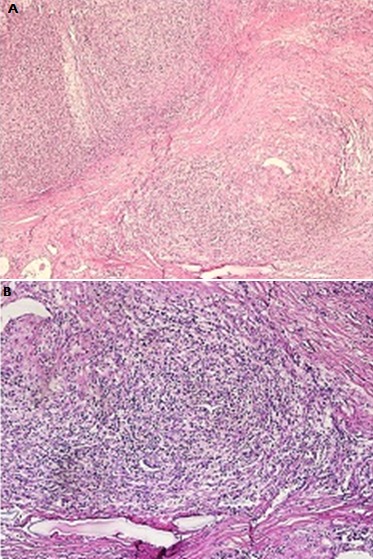
Morphologie

**Figure 4:**
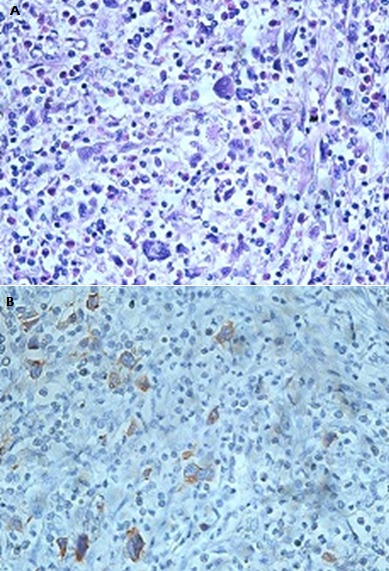
Immuno-histochimie 1

**Figure 5:**
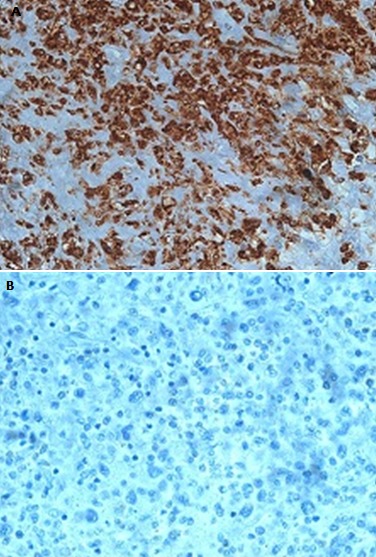
Immuno-histochimie 2

Deux mois après la chirurgie, le patient avait été adressé au service d'hématologie clinique devant le diagnostic du LH. L'examen physique objectivait un patient ECOG PS à 1, avec une reprise de la masse cervicale antérieure qui était de l'ordre de 3x4 cm sur une cicatrice propre, sans d'adénopathies périphériques palpables. Le bilan d'extension incluant une TDM cervico-thoraco-abdomino-pelvienne avait montré une masse tissulaire, hypodense homogène, occupant la loge de thyroïdectomie droite, réalisant un important effet de masse sur la trachée qui était refoulée à gauche (Figure 2); Une relecture histo-immunologique de la pièce opératoire avait montré un parenchyme thyroïdien remplacé par une prolifération tumorale faite de septa fibreux, avec expression des marqueurs CD15, CD30, sans expression des CD3; CD20 et LMP1 par les cellules tumorales (Figure 3, Figure 4, Figure 5), reconcluant pour un lymphome hodgkinien de type scléro-nodulaire. La biopsie ostéo-médullaire n'avait pas montré d'infiltration lymphomateuse; l'hémogramme, la vitesse de sédimentation, taux d'albumine et taux de LDH étaient normaux. Le bilan pré-thérapeutique était correct. Le patient avait été à postériori classé Stade IIEB selon la classification d'Ann Arbor, s'appartenant aux groupes défavorables selon les critères du German Hodgkin Lymphoma Study Group (GHSG). Sur le plan thérapeutique, le patient avait reçu 6 cycles de chimiothérapie selon le protocole ABVD avec l'Adriblastine 25mg/m², Bléomycine 10 mg/m², Vinblastine 6 mg/m² et Dacarbazine 375 mh/m² (J1 et J15), avec obtention d'une rémission complète confirmée par un TEP-scanner au FDG du corps entier réalisé en fin de traitement qui n'avait pas objectivé d'hypermétabolisme suspect notamment au niveau cervical, puis il était adressé en radiothérapie.

## Discussion

Les lymphomes de la glande thyroïde sont une entité très rare que ce soit sous forme primaire ou secondaire [9]. Les LTP représentent moins de 5% de tous les cancers de la thyroïde et jusqu'à 7% de tous les lymphomes extra-ganglionnaires [4, 10]. La majorité des lymphomes thyroïdiens sont de type non-hodgkinien avec la prédominance du lymphome diffus à grande cellules B jusqu'à 70% suivi du lymphome B de la zone marginale (MALT) [5], tandis que LH de la glande thyroïde est extrêmement rare, il représente 0.6-5% de toutes les tumeurs thyroïdiennes et 7% des lymphomes thyroïdiens [6, 11]; uniquement une trentaine de cas rapportés dans la littérature [12]. Le LTP de type hodgkinien, affecte principalement la femme (75-80% de patients) avec un âge médian de 42 ans au moment du diagnostic, comme il a été rapporté par Sa A Wang et al dans une étude de 5 cas [13] et par P. Sánchez-Velaa et al dans un tableau rassemblant les cas décrit dans la littérature [14]. Son étiologie reste inconnue, mais les lymphocytes d'une thyroïdite auto-immune, peuvent permettre une infiltration de la thyroïde (organe dépourvu de cellules lymphoïdes), par des lymphocytes [15]. De nos jours la relation entre LTP et la thyroïdite de Hashimo (TH) est bien reconnue ; la TH est une maladie auto-immune qui induit une prolifération lymphocytaire dans la thyroïde, cette relation a été aussi rapportée et décrite pour le LH thyroïdien [14], dans la série de Wang et al, 7 sur 21 sujets ont présenté une TH [13]. Notre patient avait une présentation clinique faite d'une masse cervicale thyroïdienne avec dysphagie, comme il a été rapporté dans la littérature certains patients atteints de LH thyroïdien présentaient une hypertrophie de la glande thyroïde ou une masse thyroïdienne, avec des symptômes liés à la compression ou à l'infiltration des organes du cou soit par réfléchissement des voies respiratoires ou par obstruction œsophagienne [13, 14], cette présentation clinique et aussi décrite pour les lymphomes non hodgkiniens de la thyroïde. La plupart des cas rapportés présentaient aussi des adénopathies cervicales. La forme scléro-nodulaire (SN) que présentait notre patient est la forme la plus décrite, suivie de la forme à cellularité mixte [14], cela peut être expliqué par la prédominance de cette forme et sa tendance bien reconnue dans le médiastin et la région cervicale [16]; un seul cas rapporté avait la forme lymphocytaire nodulaire du LH [12], la fibrose et la sclérose étaient aussi décrite [13]. La plupart des patients rapportés dans la littérature, comme il est le cas pour notre patient, présentaient une maladie à un stade localisé, souvent IIE dans la classification d'Ann-Arbor [14]. Cela implique un bon pronostic en cas de traitement précoce. Actuellement, l'objectif est d´essayer de réduire la toxicité du traitement, tout en maintenant son efficacité. Les LTP hodgkiniens sont traités comme pour les LH nodaux, par association de polychimiothérapie et radiothérapie [13, 17], notre patient avait reçu 6 cycles de chimiothérapie de type ABVD et une radiothérapie.

## Conclusion

Malgré sa rareté et sa localisation inhabituelle, le lymphome hodgkinien de la glande thyroïde, doit être connu et pris en compte dans le diagnostic différentiel des tumeurs thyroïdiennes. Un diagnostic et prise en charge précoces permettent d'obtenir des résultats thérapeutiques favorables, avec des taux de guérison de plus 80%.

## Conflits d’intérêts

Les auteurs ne déclarent aucun conflit d'intérêts.
